# Prooxidant Effects of Verbascoside, a Bioactive Compound from Olive Oil Mill Wastewater, on *In Vitro* Developmental Potential of Ovine Prepubertal Oocytes and Bioenergetic/Oxidative Stress Parameters of Fresh and Vitrified Oocytes

**DOI:** 10.1155/2014/878062

**Published:** 2014-02-25

**Authors:** M. E. Dell'Aquila, L. Bogliolo, R. Russo, N. A. Martino, M. Filioli Uranio, F. Ariu, F. Amati, A. M. Sardanelli, V. Linsalata, M. G. Ferruzzi, A. Cardinali, F. Minervini

**Affiliations:** ^1^Section of Veterinary Clinics and Animal Production, Department of Emergency and Organ Transplantation (DETO), University of Bari “Aldo Moro”, Strada Provincial Casamassima Km 3, Valenzano, 70010 Bari, Italy; ^2^Obstetric and Gynecological Section, Department of Veterinary Medicine, Via Vienna 2, 07100 Sassari, Italy; ^3^Department of Basic Medical Sciences, Neurosciences and Sense Organs, University of Bari “Aldo Moro”, Piazza Giulio Cesare, 70124 Bari, Italy; ^4^Institute of Translational Pharmacology (IFT), National Research Council (CNR), Via Fosso del Cavaliere No. 100, 00133 Rome, Italy; ^5^Institute of Sciences of Food Production (ISPA), National Research Council (CNR), Via G. Amendola 122/O, 70126 Bari, Italy; ^6^Department of Food Science, 745 Agriculture Mall Drive, West Lafayette, IN 47907, USA

## Abstract

Verbascoside (VB) is a bioactive polyphenol from olive oil mill wastewater with known antioxidant activity. Oxidative stress is an emerging problem in assisted reproductive technology (ART). Juvenile ART is a promising topic because, in farm animals, it reduces the generation gap and, in human reproductive medicine, it helps to overcome premature ovarian failure. The aim of this study was to test the effects of VB on the developmental competence of ovine prepubertal oocytes and the bioenergetic/oxidative stress status of fresh and vitrified oocytes. In fresh oocytes, VB exerted prooxidant short-term effects, that is, catalase activity increase and uncoupled increases of mitochondria and reactive oxygen species (ROS) fluorescence signals, and long-term effects, that is, reduced blastocyst formation rate. In vitrified oocytes, VB increased ROS levels. Prooxidant VB effects in ovine prepubertal oocytes could be related to higher VB accumulation, which was found as almost one thousand times higher than that reported in other cell systems in previous studies. Also, long exposure times of oocytes to VB, throughout the duration of *in vitro* maturation culture, may have contributed to significant increase of oocyte oxidation. Further studies are needed to identify lower concentrations and/or shorter exposure times to figure out VB antioxidant effects in juvenile ARTs.

## 1. Introduction

Verbascoside (VB) or acteoside is a phenylpropanoid glycoside structurally characterized by the caffeic acid linked by a **β**-(D)-glucopyranoside to 4,5-hydroxyphenylethanol (hydroxytyrosol) bound through ester and glycosidic links, with a rhamnose in sequence (1–3) to the glucose molecule. Verbascoside can be extracted, as other phenolic compounds, from primary plants, such as olives, but it is also present in a good amount in olive oil mill wastewater (OMWW), a phytotoxic material discarded during olive oil production [1]. For long time, OMWW has been regarded as a hazardous waste with negative impact on the environment and an economic burden on olive oil industry. However, this view has recently changed to recognise OMWW as a potential low cost starting material rich in bioactive compounds, particularly phenolic compounds, which can be used as natural antioxidants in various systems [2]. Anti-inflammatory activity of VB has also been demonstrated by *in vitro* tests performed on cell cultures of primary human keratinocytes [3], on the inflammatory bowel disease in a mouse model [4], and on glioma cell line (G6) [5]. Other studies demonstrate that VB prevents reactive oxygen species (ROS) related damages in different ways, such as by interfering with initial ROS generating reactions, by scavenging the free oxygen molecules required to begin ROS production, by chelating metals that speed up oxidative processes, and by inducing glutathione transferase (GST) activity [6, 7]. In a recent study, VB antioxidant activity has been evidenced by using two different bioassays: a cell-free system, based on *in vitro* copper (Cu^+^) induced low density lipoproteins (LDL) peroxidation, and an *in vitro* cultured cell system (HT-29 intestinal cell line), based on the observation of VB effects on intracellular ROS levels [2].

Oxidative stress occurs if disequilibrium between ROS production and the antioxidative capacity of the cell takes place [8]. Reactive oxygen species, such as superoxide anion (O_2_^−^) inactivated by the superoxide dismutase (SOD), hydrogen peroxide (H_2_O_2_) inactivated by catalase (CAT), and hydroxyl radical (OH^−^) inactivated by glutathione peroxidase (GPX), are oxygen-derived molecules which have the ability to react with any molecule and modify it oxidatively, resulting in structural and functional alterations. They are not just damaging by-products of respiration but, at low concentration, are also important molecules for cell signalling within intracellular compartments [9]. Reactive oxygen species have also been implicated in the aetiology of some forms of female infertility [10]. The effects of ROS on oocytes can be clearly evidenced in assisted reproductive techniques (ARTS) setting, due to the reduction or lack in the culture environment of physiological defence mechanisms available in the female reproductive tract, such as the follicular and oviductal fluids and cumulus oophorus matrix [11].

A major source for ROS production is the mitochondrion, in which they are produced during oxidative phosphorylation [12–14]. Mitochondria are essential organelles in all eukaryotic cell systems, as the powerhouse to provide ATP for a multitude of cellular processes. They are the hub of metabolic pathways, primary sources of ROS, regulators of apoptosis as well as signal transduction regulators, and buffers of intracellular calcium [15, 16]. Increasing evidences show the essential role of mitochondria as determinants for developmental competence for human and mammalian oocytes [17–19]. Mitochondrial (mt) dysfunctions or abnormalities may compromise developmental processes, by inducing chromosomal segregation disorders, maturation and fertilization failures, or embryonic cell fragmentation resulting in mitochondria-driven apoptosis. Mitochondrial distribution and activity and oxidative stress status have been analyzed by confocal laser scanning microscopy (CLSM) in oocytes of several species [20].

*In vitro* embryo production (IVP) using oocytes derived from prepubertal subjects in conjunction with *in vitro* embryo transfer, termed as juvenile *in vitro* embryo transfer (JIVET), is pursued with the aim of increasing the rate of genetic gain through a reduction of the generation gap [21, 22]. Moreover, this technology could be employed in human ART to preserve female fertility of young adolescent or paediatric premenarcheal patients affected by cancer or by different forms of premature ovarian failure [23–25]. A widely used animal model for the JIVET research field is the ovine species, but despite considerable research and great interest in this technology, the efficiency of JIVET remains low mainly due to reduced meiotic and developmental competence of prepubertal oocytes [22, 26]. In addition to biological concerns, JIVET technology has the need of oocyte cryopreservation which still strictly affects prepubertal oocyte fertilization and developmental competence. Among cryopreservation procedures, vitrification, that is, the solidification of a solution at low temperature, a process achieved by a combination of a high concentration of cryoprotectants and an extremely high cooling rate, is an alternative approach to slow freezing, because it avoids the formation of ice crystals in the intracellular and extracellular spaces [27–31]. Few papers have been published to date on vitrification of prepubertal farm animal oocytes [32–34] as well as in human pediatric oocytes [35]. Also, few studies on mt bioenergetic potential of fresh or vitrified prepubertal oocytes have been reported in farm animals to date [36, 37] but no studies in humans. Lastly, no data on oxidative stress parameters of prepubertal oocytes are available in the literature in any species. In this context, studies aimed to evaluate the potentially antioxidant activity of natural bioactive compounds, such as phenolic compounds and polyphenols, present in plant-derived by products would be beneficial to improve cryopreservation and *in vitro* culture protocols of prepubertal oocytes.

The aim of the present study was to test the effects of VB, added during *in vitro* maturation (IVM) culture, on oocyte maturation, cleavage, and blastocyst formation rates of fresh oocytes and on bioenergetic/oxidative status of fresh and vitrified oocytes. Moreover, the stability of VB in the IVM culture system and its absorptive potential by the oocyte-cumulus complex (OCC) were also assessed.

## 2. Materials and Methods

All chemicals were purchased from Sigma-Aldrich (Milano, Italy) unless otherwise indicated.

### 2.1. Verbascoside Extraction and Purification and HPLC Analysis

Verbascoside was recovered from OMWW following the protocol described by Cardinali et al. [1]. Verbascoside was purified by low-pressure gel filtration chromatography on a Sephadex LH-20 column (40 cm × 1.6 cm, Pharmacia) and quantified by High Performance Liquid Chromatography with diode array detection (HPLC-DAD) analysis. Analytical-scale HPLC analyses of VB were performed using a Thermo Scientific HPLC Spectra System equipped with a P2000 gradient pump, an SCM1000 vacuum membrane degasser, a UV6000LP diode array UV-VIS detector, an AS3000 autosampler, and ThermoQuest software. The ChromQuest Chromatography data system 4.1 version was used for spectra and data processing. For the HPLC analysis, an analytical Phenomenex (Torrance, CA) Luna C18 (5 *μ*m) column (4.6 × 250 mm) was used throughout this work. The solvent system consisted of (A) methanol and (B) acetic acid/water (5 : 95, v/v). The elution profile was 0–25 min 15–40% A in B (linear), 25–30 min 40% A in B (isocratic), 30–45 min 40−63% A in B (linear), 45−47 min 63% A in B (isocratic), and 47–51 min 63–100% A in B (linear). The flow rate was 1 mL/min. Samples of 25 *μ*L were applied to the column by means of a 25 *μ*L loop valve.

### 2.2. Oocyte Collection

Ovaries from juvenile (less than 6 months of age) sheep, recovered at two local slaughterhouses, located at a maximum distance of 20 km (30 min) from the laboratory, were processed by the slicing procedure [38]. Only OCCs with intact cumulus cells layers and a homogeneous cytoplasm were selected.

### 2.3. Oocyte *In Vitro* Maturation

*In vitro* maturation was performed following the procedures currently in use in the two laboratories (Bari and Sassari). Experiments performed in Sassari aimed to assess the effect of VB exposure during IVM on oocyte nuclear maturation, cleavage and blastocyst developmental rates and on the viability of vitrified/warmed metaphase II (MII) oocytes. Oocytes were cultured in the conditions described by Bogliolo et al. (Medium 1; [39]). *In vitro* maturation was performed in TCM-199, buffered with 249 mM sodium bicarbonate and supplemented with 1.8 *μ*M sodium pyruvate, 10% Oestrus Sheep Serum (OSS), 0.1 IU/mL FSH, 0.1 IU/mL LH, 100 *μ*M cysteamine, 0.1 g/L penicillin, and 0.1 g/L streptomycin. Oocyte-cumulus complexes were cultured in four-well Petri dishes (Nunclon; Nalge Nunc International, Roskilde, Denmark) covered with 300 *μ*L preequilibrated mineral oil for 24 h under 5% CO_2_ in air at 38.5°C. Experiments conducted in Bari aimed to perform confocal microscopy and biochemical analyses of bioenergetic/oxidative status of fresh and vitrified/warmed MII oocytes exposed to VB during IVM culture and to perform the stability and uptake assays. Oocytes were cultured as reported by Martino et al. [40] with some modifications (Medium 2). Medium TCM-199 with Earle's salts, buffered with 4.43 mM 4-(2-hydroxyethyl)-1-piperazineethanesulfonic acid (HEPES) and 33.9 mM sodium bicarbonate and supplemented with 0.1 g/L L-glutamine, 2 mM sodium pyruvate, 2.92 mM calcium-L-lactate pentahydrate (Fluka 21175; Serva Feinbiochem), 50 *μ*g/mL gentamicin, 10% (v/v) Fetal Calf Serum (FCS), 10 *μ*g/mL ovine FSH and 20 *μ*g/mL ovine LH, and 1 *μ*g/mL 17 beta estradiol was used. Oocyte-cumulus complexes were placed in 400 *μ*L of medium/well of a four-well dish (Nunc Intermed, Roskilde, Denmark) covered with preequilibrated lightweight paraffin oil and cultured for 24 h at 38.5°C under 5% CO_2_ in air. After IVM culture, oocytes underwent cumulus and corona cells removal by incubation in TCM-199 with 20% FCS containing 80 IU hyaluronidase/mL and aspiration in and out of finely drawn glass pipettes. Verbascoside stock solution (100 *μ*M) was prepared on the day of use by dissolving VB in distilled water and added in the maturation medium for the whole duration of IVM culture at three increasing concentrations (1.03, 2.06, and 4.11 *μ*M) which were reported as being effective as ROS scavenger [2].

### 2.4. Oocyte Vitrification

Oocyte vitrification was performed with the method of minimum essential volume (MEV), as described by Bogliolo et al. [41]. Briefly, groups of five MII oocytes were initially equilibrated at 38.5°C for 1 min in holding medium (HM) consisting of 20 mM HEPES-buffered TCM-199 supplemented with 20% (v/v) FCS. After equilibration, the oocytes were incubated in 10% (v/v) ethylene glycol (EG) + 10% (v/v) dimethylsulfoxide (DMSO) in HM for 30 sec and then transferred to 20% (v/v) EG and 20% (v/v) DMSO and 0.25 M sucrose in HM for 20 s. The oocytes were then loaded on cryotops (Kitazato Ltd., Tokyo, Japan) and immediately plunged into liquid nitrogen (LN_2_) for storage. For warming, cryotops were directly inserted in HM supplemented with 1.25 M sucrose for 1 min. Oocytes were transferred into HM at decreasing sucrose concentrations (0.62 M and 0.31 M) and then washed in HM. Warmed oocytes were cultured for 1 h in TCM-199 supplemented with 10% OSS and their viability was assessed by observation under a Nikon SMZ 1500 stereomicroscope (60–110x magnification).

### 2.5. *In Vitro* Fertilization (IVF) and *In Vitro* Embryo Development

As described by Berlinguer et al. [42], *in vitro* matured oocytes were fertilized in Synthetic Oviductal Fluid (SOF, [43]) + 2% OSS + 1 *μ*g/mL heparin + 1 *μ*g/mL hypotaurine for 22 h at 38.5°C and under a 5% CO_2_, 5% O_2_, and 90% N_2_ atmosphere in four-well Petri dishes with frozen-thawed spermatozoa selected by swim-up technique (1 × 10^6^ spermatozoa/mL^−1^). Presumptive zygotes were cultured for 8 days in four-well Petri dishes in SOF + essential and nonessential amino acids at oviductal concentration [44] + 0.4% Bovine Serum Albumin (BSA) under mineral oil, in maximum humidified atmosphere with 5% CO_2_, 5% O_2_, and 90% N_2_ at 38.5°C.

### 2.6. Nuclear Chromatin Evaluation of Oocytes and Embryos

To evaluate nuclear chromatin, at the end of IVM and IVP, oocytes and embryos were stained with 2.5 *μ*g/mL Hoechst 33258 in 3 : 1 (v/v) glycerol/PBS and mounted on microscope slides covered with cover slips, sealed with nail polish, and kept at 4°C in the dark until observation. Oocytes were evaluated in relation to their meiotic stage under an epifluorescence microscope (Nikon Eclipse 600, 400x magnification) equipped with the B-2 A (346 nm excitation/460 nm emission) filter, as the germinal vesicle (GV), metaphase to telophase I (MI to TI), MII with 1st polar body (PB) extruded and degenerated [45]. Embryos were classified as normal when the presence of a regular shaped nucleus inside each blastomere was observed [39, 46].

### 2.7. Oocyte Mitochondria and ROS Staining

After IVM, additional oocytes showing the 1st PB extruded (MII stage oocytes) were washed three times in PBS with 3% BSA and incubated for 30 min in the same medium containing 280 nM MitoTracker Orange CMTM Ros (Molecular Probes M-7510, Oregon, USA) at 38.5°C under 5% CO_2_ [20, 40, 47]. The cell-permeant probe contains a thiol-reactive chloromethyl moiety. Once the MitoTracker probe accumulates in the mitochondria, it can react with accessible thiol groups on peptides and proteins to form an aldehyde-fixable conjugate. This cell-permeant probe is readily sequestered only by active mitochondria [48, 49]. The organelle specificity of the probe was assessed, as reported by Valentini et al. [50], in control oocytes which were imaged after incubation in MitoTracker Orange and further incubation for 5 min in the presence of 5 *μ*M of the mt membrane potential (Delta Psi) collapsing uncoupler carbonyl cyanide 3-chloro phenyl hydrazone (CCCP; Molecular Probes), which inhibits mt respiratory activity, thus reducing fluorescence intensity. After incubation with mt probe, oocytes were washed three times in PBS with 0.3% BSA and incubated for 15 min in the same media containing 10 *μ*M 2′,7′-dichlorodihydrofluorescein diacetate (H_2_DCF-DA) [51, 52] in order to detect and localize intracellular sources of ROS. The principle underlying this procedure may be described briefly as follows: nonionized H_2_DCF-DA is membrane permeant and therefore is able to diffuse readily into cells. Once within the cell, the acetate groups are hydrolysed by intracellular esterase activity forming 2′,7′-dichlorodihydrofluorescein (H_2_DCF) which is polar and thus trapped within the cell. H_2_DCF fluoresces when it is oxidized by H_2_O_2_ or lipid peroxides to yield 2′,7′-dichlorofluorescein (DCF). The level of DCF produced within the cells is related linearly to that of peroxides present and thus its fluorescent emission provides a measure of the peroxide levels [51]. After incubation, oocytes were washed three times in prewarmed PBS without BSA and fixed overnight at 4°C with 2% paraformaldehyde solution in PBS.

### 2.8. Assessment of Oocyte Mitochondrial Distribution Pattern and Intracellular ROS Localization

For mt distribution pattern evaluation, MII oocytes were selected among those having regular ooplasmic size and texture (no vacuoles). Oocytes were observed at 600x magnification in oil immersion with Nikon C1/TE2000-U laser scanning confocal microscope. A helium/neon laser ray at 543 nm and the G-2 A filter (551 nm exposure and 576 nm emission) were used to point out the MitoTracker Orange CMTM Ros. An argon ions laser ray at 488 nm and the B-2 A filter (495 nm exposure and 519 nm emission) were used to point out the DCF. Scanning was conducted with 25 optical series from the top to the bottom of the oocyte with a step size of 0.45 *μ*m to allow three-dimensional distribution analysis. General criteria for oocyte mt patterns definition were adopted on the basis of previous studies in other species [20, 40, 47, 50]. Homogeneous/even distribution of small mt aggregates throughout the cytoplasm was considered as indication of immature cytoplasmic condition, whereas heterogeneous/uneven distribution of small and/or large aggregates within the cytoplasm indicated a metabolically active ooplasm. In particular, accumulation of mitochondria in the peripheral cytoplasm and around the nucleus was considered as aspects of the developmental program of cytoplasmic maturation [53]. Oocytes showing irregular distribution of large mt clusters unrelated to the specific cell compartments were classified as abnormal. To our knowledge, few studies are reported to date on intracellular ROS localization in mammalian oocytes. Recent studies performed in mouse [54], equine [47], and ovine [40] oocytes from adult females reported mt/ROS colocalization as a biomarker of healthy oocytes.

### 2.9. Quantification of MitoTracker Orange CMTM Ros and DCF Fluorescence Intensity

Measurements of fluorescence intensities were performed in MII oocytes having either heterogeneous (perinuclear and/or pericortical) or homogeneous (small aggregates) mt distribution pattern. Oocytes showing abnormal mt distribution pattern were excluded from this analysis. In each individual oocyte, MitoTracker and DCF fluorescence intensities were measured at the equatorial plane, as in previous studies from our unit performed in human [55] and animal oocytes [20, 40, 47, 50], with the aid of the EZ-C1 Gold Version 3.70 image analysis software platform for Nikon C1 (Nikon Instruments) confocal microscope. A circle of an area = 100 in diameter (arbitrary value) was drawn to measure only the cytoplasmic area (512 × 512 pixels). The fluorescence intensity encountered within the programmed scan area was recorded and plotted against the conventional pixel unit scale (0–255). Fluorescence intensity was expressed as arbitrary densitometric units (ADU). Parameters related to fluorescence intensity were maintained at constant values for all measurements. In detail, images were taken under fixed scanning conditions with respect to laser energy, signal detection (gain), and pinhole size.

### 2.10. Oocyte Mitochondria/ROS Colocalization Analysis

Colocalization analysis of mitochondria and ROS was performed by using the EZC1 Gold Version 3.70 software. Degree of colocalization was reported as Pearson's correlation coefficient quantifying the overlap degree between MitoTraker Orange CMTM Ros and DCF fluorescence signals [40].

### 2.11. Measurement of the Total CAT Activity in Single Oocytes

Additional MII oocytes were washed in SB buffer (TRIS/HCl 60 mM pH 6.8, Glycerol 40%) and individually solubilized for one hour at 4°C in the presence of 1.0% Triton X-100. The protein concentration was assessed by the method of Bradford [56]. Each test was performed on 7 *μ*g of proteins from a single solubilized oocyte. The CAT activity was determined following the Beers and Sizer method [57]. A spectrophotometer Varian Cary WinUV50 was used to follow the absorbance decrease at 240 nm in the presence of H_2_O_2_ for time unit, in potassium-phosphate 50 mM, EDTA 1 mM pH 7.0, at 25°C, in a final volume of 1 mL. After determining the baseline with the measure mixture, 3.5 mM H_2_O_2_ was added. Reaching stability, the reactions with protein extract started. Since H_2_O_2_ molar extinction coefficient (*ε* = 40 M^−1^ ∗ cm^−1^) is known, CAT activity was calculated as *μ*moles of H_2_O_2_ converted in H_2_O per minute. Specific activities were calculated by dividing enzymatic activities per total protein concentration expressed in mg.

### 2.12. Measurement of the Total SOD Activity in Single Oocytes

Additional MII oocytes were washed in SB buffer (TRIS/HCl 60 mM pH 6.8, Glycerol 40%) and individually solubilized for one hour at 4°C in the presence of 1.0% Triton X-100. The protein concentration was assessed by the method of Bradford [56]. Each test was performed on 7 *μ*g of proteins of a single solubilized oocyte. SOD activity was determined with the Fluka analytical assay kit using a multiplate reader Victor X, Perkin Elmer at **λ** = 440 nm. Total SOD activity (SOD, EC 1.15.1.1) was assayed by its ability to inhibit the reduction of a novel tetrazolium salt, WST-1[2-(4-Iodophenyl)-3-(4-nitrophenyl)-5-(2,4-disulfophenyl)-2H-tetrazolium, monosodium salt] by superoxide anions generated with the xanthine/xanthine oxidase method [58, 59]. One unit of SOD activity was defined as the amount of the enzyme causing half maximum inhibition of WST-1 reduction. It was measured with high sensitivity (0.01 pmol) and expressed as U/mg proteins. A nine-point standard curve was routinely included in each assay.

### 2.13. Verbascoside Stability in Oocyte-Free Culture Medium and Its Uptake by the OCC

Verbascoside stability in the IVM culture medium was assessed in the same conditions as that used in IVM experiments. In particular, VB (10 *μ*M) was added to the maturation medium in the absence of oocytes for 24 h at 38.5°C. After 0, 4, 12, and 24 h, aliquots of the mixed solutions were analyzed by HPLC-DAD as described in HPLC analysis section. For accumulation experiments, OCCs were cultured in maturation medium containing 100 *μ*M VB. Oocytes were then incubated at 38.5°C for 30 and 60 min. Following incubation, media were aspirated and stored at −80°C until analysis; oocytes were washed 3 times with PBS (pH 7.4) then were collected and stored at −80°C until analysis. Protein values for each tested oocyte pool (*n* = 2 pools of 36 OCCs each) were assessed by Bio-Rad protein assay method [56] and were found to be 0.504 ± 0.05 mg of total protein/pool. Verbascoside was extracted from sonicated OCCs with 3 mL of ethyl acetate (0.01% butylated hydroxytoluene) for three times. Ethyl acetate layers were pooled, dried under vacuum, and resolubilized in 300 *μ*L of HPLC mobile phase. HPLC analysis was performed following the protocol as described before.

### 2.14. Statistical Analysis

Oocyte nuclear maturation rates, cleavage and blastocyst developmental rates, and the percentages of oocytes showing different mt and intracellular ROS distribution patterns were compared between treated and control groups by Chi-square test with the Yates correction for continuity. The effects of VB on nuclear maturation rates were also assessed by multifactor ANOVA, taking into account the use of the two IVM media. Fisher's exact test was applied in all cases in which at least one cell contained a value less than 5. For confocal quantification analysis of mt activity and intracellular ROS levels, the least-square means of the dependent variable (MitoTracker CMTM Ros and DCF fluorescence intensity) were calculated in examined samples and the statistical significance of the least-square means between treated and control groups was calculated by one-way ANOVA followed by Multiple Comparison Dunn's method (SigmaPlot software). For mt/ROS colocalization, mean values of Pearson's correlation coefficient were compared between treated and control groups by one-way ANOVA followed by Multiple Comparison Dunn's method (SigmaPlot software). CAT and SOD activities were compared between treated and control groups by Student's *t* test. Differences with *P* < 0.05 were considered as statistically significant.

## 3. Results

### 3.1. Verbascoside Extraction and Purification and HPLC Analysis

Verbascoside was purified from OMWW by using a laboratory scale system to remove solid particles from the fluid and to fractionate molecules with different molecular weight. From the two permeate fractions obtained, microfiltrate (MF) and ultrafiltrate (UF), UF was considered to be around 50% of the initial volume of OMWW (5 L of UF from 10 L of OMWW). This fraction was subsequently separated by low-pressure gel filtration chromatography on a Sephadex LH-20 column to give a highly purified VB fraction. The concentration of VB obtained was on average 3.25 mg/30 mL of UF giving a final yield of around 54 mg of VB from each liter of OMWW. Purification degree for VB, calculated on peak area, was around 95% as demonstrated by HPLC analysis.

### 3.2. Verbascoside Did Not Affect the Nuclear Maturation Rate of Fresh Prepubertal Lamb Oocytes

One thousand and twenty-nine oocytes were recovered, 850 of which were surrounded by a healthy cumulus and were selected for IVM culture. Oocytes were cultured in IVM medium 1 or in IVM medium 2. For each medium, five consecutive trials were performed. In Table 1, separated and pooled data obtained with both culture media are presented. Verbascoside did not affect the maturation rate of prepubertal lamb oocytes neither when added in medium 1 nor when added in medium 2. No effects were also noticed in pooled data of oocytes matured in medium 1 + medium 2.

On the other hand, within each medium and at specific tested VB concentrations (2.06 and 4.11 *μ*M), an indirect beneficial influence of medium 1 composition on the VB effects on oocyte meiosis resumption and progression to the MII stage was noticed. In oocytes cultured in presence of 2.06 *μ*M VB, a significantly higher rate of those cultured in medium 1 resumed meiosis (significantly lower rate of GV oocytes; *P* < 0.001) and reached the metaphase stages (*P* < 0.001) compared with those cultured in medium 2. In oocytes cultured in presence of 4.11 *μ*M, a significantly lower rate of GV oocytes (*P* < 0.001) was found and the maturation rate to the MII stage tended to be higher (*P* = 0.054) compared with those cultured in medium 2. Thus, possibly, the VB effects may differ in oocytes primed by different media. The multifactor ANOVA analysis confirmed Chi-square based significances.

### 3.3. Verbascoside Impaired the Blastocyst Formation Rate of Fresh Prepubertal Lamb Oocytes

Nine hundred MII oocytes were used for IVF and *in vitro* embryo culture experiments. Early cleavage and blastocyst formation rates were recorded as resumed in Table 2. After IVF, for each condition, the number of cleaved oocytes at 24 and 30 hours after fertilization and the number of embryos reaching the blastocyst stage at day 7 and day 8 were recorded. No significant differences in the early cleavage rate were found between VB-treated and control groups. Significant reductions of the blastocyst formation rate were found in the group of oocytes exposed to 2.06 and 4.11 *μ*M VB compared with controls (Table 2; *P* < 0.05).

### 3.4. Verbascoside Did Not Affect mt Distribution Pattern and Intracellular ROS Localization of Fresh MII Stage Prepubertal Lamb Oocytes

One hundred and thirteen fresh oocytes matured *in vitro* and found at the MII stage were analyzed. No significant effects of VB on mt distribution pattern of fresh prepubertal lamb MII stage oocytes was found (Table 3). Intracellular ROS localization was also unaffected by VB and data concerning this parameter corresponded to data of mt distribution pattern presented in Table 3.

### 3.5. Verbascoside, at the Highest Tested Concentration, Increased mt Activity and ROS Levels in MII Fresh Prepubertal Lamb Oocytes

**Figure 1:**
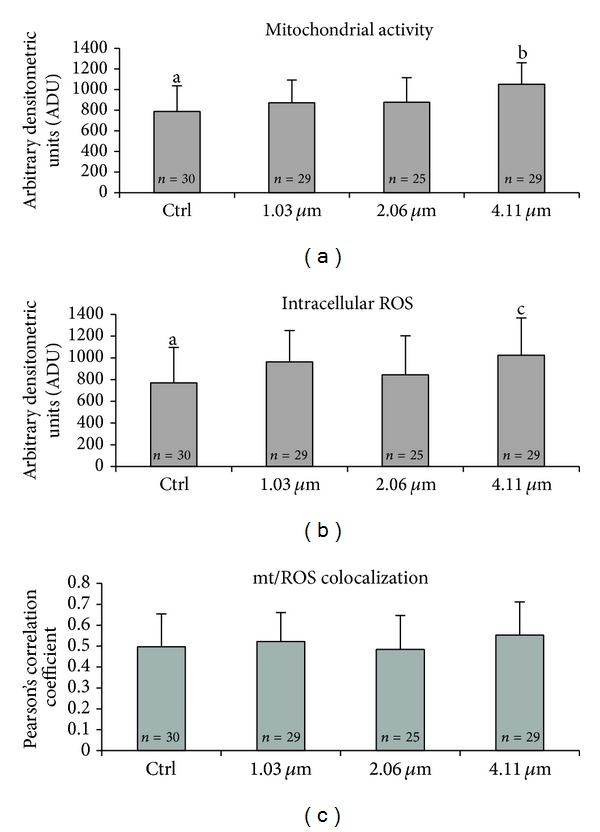
Dose response curve of the *in vitro* effects of VB on mt activity and intracellular ROS levels in single fresh prepubertal lamb MII stage oocytes, expressed as MitoTracker Orange CMTM Ros (panel (a)) and DCF (panel (b)) fluorescence intensities. Oocytes treated with 4.11 *μ*M VB showed significantly higher mt activity and intracellular ROS levels compared with controls. Values are expressed as ADU (arbitrary densitometric units). Pearson's correlation coefficients of MitoTracker Orange CMTM Ros and DCF fluorescent labelling colocalization of treated versus control MII oocytes (panel (c)). Numbers of analyzed oocytes per group are indicated at the bottom of each histogram. (a) One-way ANOVA: a, b: *P* < 0.05; (b) one-way ANOVA: a, c: *P* < 0.001; and (c) one-way ANOVA: NS (not significant).

**Figure 2:**
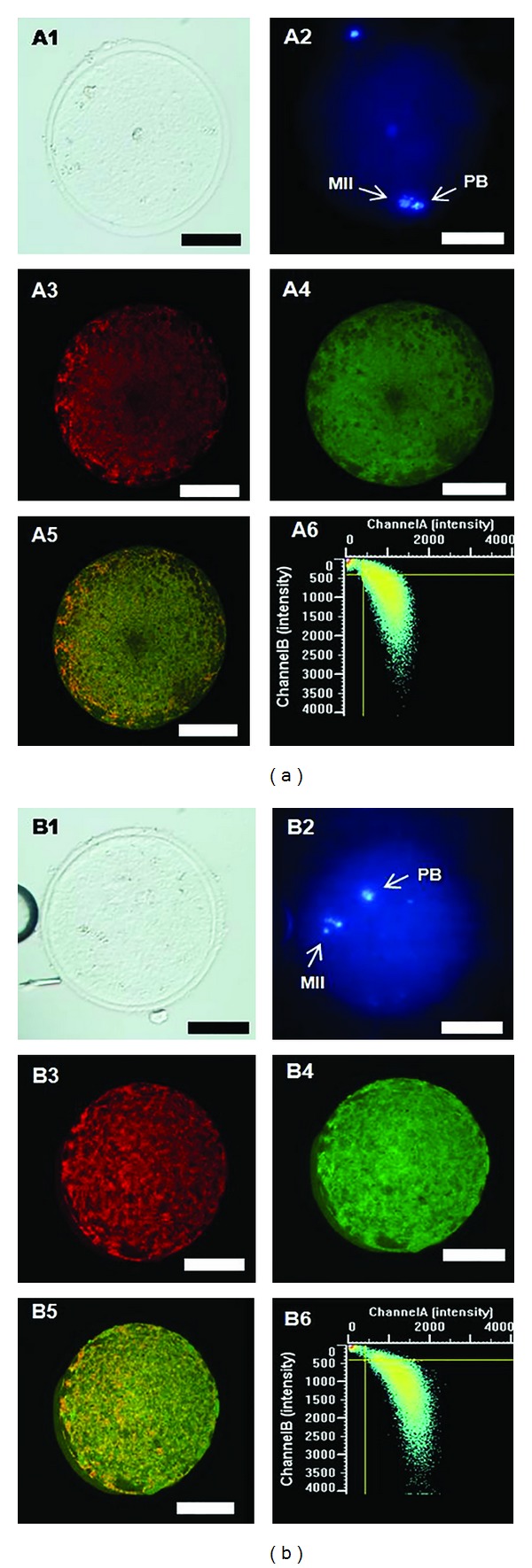
Mitochondrial distribution pattern and activity, intracellular ROS localization and levels, and mt/ROS colocalization of a representative prepubertal lamb control MII oocyte (panel (a)) and a MII oocyte representative of those cultured in presence of 4.11 *μ*M VB (panel (b)). Both oocytes show heterogeneous mt pattern. For each culture condition, corresponding bright-field (A1, B1), UV light (A2, B2), and confocal laser scanning images of mt distribution pattern (A3, B3), intracellular ROS localization (A4, B4), mt/ROS merge (A5, B5), and colocalization scatterplot graph (A6, B6) are shown. Increased mt activity, expressed as MitoTracker Orange CMTM Ros fluorescence intensity, and intracellular ROS levels, expressed as DCF fluorescent intensity (B4), can be seen in the oocyte exposed to 4.11 *μ*M VB compared with the control oocyte (B3 versus A3 and B4 versus A4, resp.). Scale bars represent 50 *μ*M.

A significant increase in mt activity was found in oocytes treatment groups exposed to the highest VB concentration (4.11 *μ*M) compared with controls (Figure 1(a), *P* < 0.05). No variations were found, at the lower concentrations assessed. As well, in the 4.11 *μ*M VB exposed group, a significant increase in intracellular ROS levels was found compared with controls (Figure 1(b), *P* < 0.001). The mt/ROS colocalization, expressed as Pearson's correlation coefficient, was not affected by VB addition (Figure 1(c), NS).  Figure 2 shows mt distribution pattern and activity, intracellular ROS localization and levels, and mt/ROS colocalization of a representative control MII oocyte (Figure 2(a)) and a MII oocyte representative of those cultured in presence of 4.11 *μ*M VB (Figure 2(b)). Increased MitoTracker and DCF fluorescence intensities, indicating mt activity and ROS levels, are evident in the VB-treated oocyte compared with the control one.

### 3.6. Verbascoside Affected CAT but Not Total SOD Activity in MII Fresh Prepubertal Lamb Oocytes

**Figure 3:**
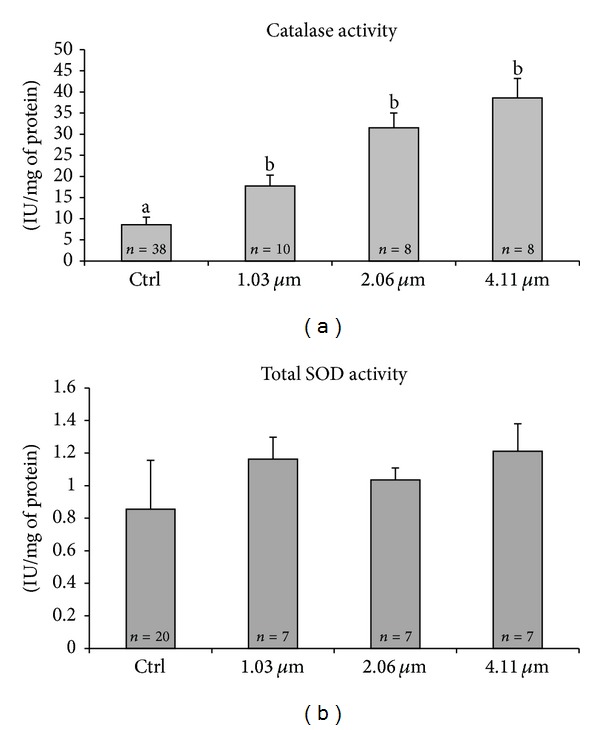
Catalase (CAT) and total superoxide dismutase (SOD) activities in individual prepubertal lamb MII oocytes matured *in vitro* in presence of VB. Numbers of analyzed oocytes per group are indicated at the bottom of each histogram. Values are expressed as IU/mg protein. Treatment with VB induced a significant increase in CAT activity compared to controls (panel (a); Student's *t*-test: a, b; *P* < 0.001). Total SOD activity did not vary between treated and control groups (panel (b); Student's *t*-test: NS).

Scavenging enzymes, CAT and SOD, activities were evaluated on fresh MII oocytes. CAT activity assay was conducted on individual oocytes (*n* = 64) that showed the 1st PB extruded after IVM. For SOD activity assay, 41 individual MII oocytes were evaluated. Results are reported in Figure 3. Significant increase of CAT activity in VB-treated groups was observed compared with controls (Figure 3(a); *P* < 0.05). On the contrary, SOD activity was not affected by VB addition and constant levels were found at any tested concentration (Figure 3(b), NS).

### 3.7. Verbascoside Did Not Affect Oocyte Viability in MII Vitrified/Warmed Prepubertal Lamb Oocytes

Oocytes cultured *in vitro* in presence of different VB concentrations and found at the MII stage were vitrified/warmed and analyzed for cell viability. Oocyte viability assessment, expressed as the percentage of oocytes showing zona pellucida and oolemma integrity and absence of ooplasmic cyto-fragmentation, was conducted on 209 vitrified/warmed oocytes. No significant effects of VB on oocyte viability after vitrification/warming were observed (Table 4).

### 3.8. Verbascoside Did Not Affect mt Distribution Pattern and ROS Intracellular Localization in MII Vitrified/Warmed Prepubertal Lamb Oocytes

Oocytes cultured *in vitro* in presence of different VB concentrations and found at the MII stage were vitrified/warmed and analyzed for confocal parameters expressing the bioenergetic/oxidative status. Sixty-six vitrified/warmed oocytes were analyzed. Verbascoside did not affect the mt distribution pattern in MII vitrified/warmed prepubertal lamb oocytes (Table 5). Intracellular ROS localization also did not vary upon VB addition in vitrified samples and data concerning this parameter corresponded to those presented in Table 5, as previously observed for fresh oocytes.

### 3.9. Verbascoside Did Not Affect mt Activity but Increased ROS Levels in MII Vitrified/Warmed Prepubertal Lamb Oocytes

**Figure 4:**
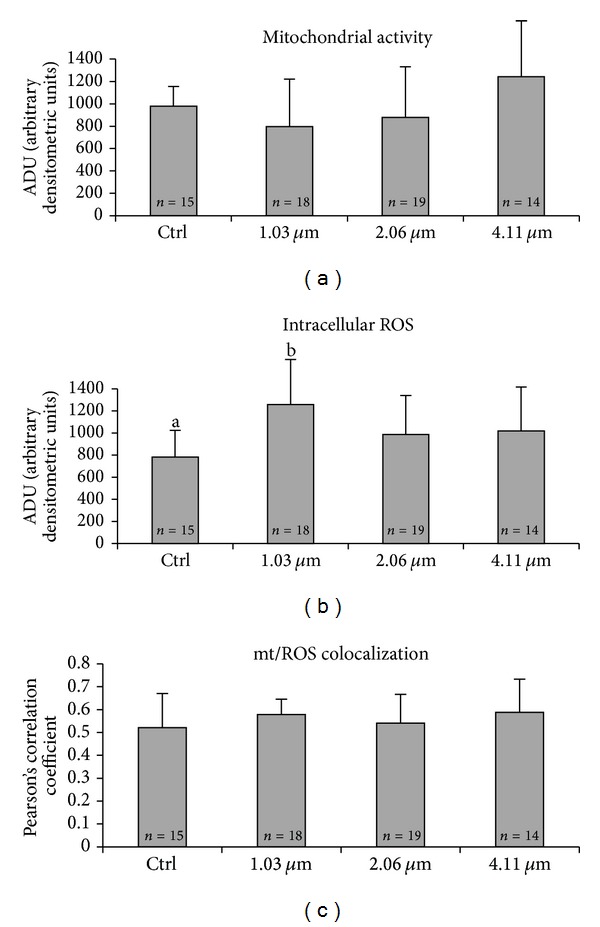
Dose response curve of the *in vitro* effects of VB, added during IVM culture, on mt activity and intracellular ROS levels in single prepubertal lamb MII stage oocytes vitrified/warmed, expressed as MitoTracker Orange CMTM Ros (panel (a)) and DCF (panel (b)) fluorescence intensities. Oocytes treated with 1.03 *μ*M VB showed significantly higher intracellular ROS levels compared with controls. Values are expressed as ADU (arbitrary densitometric units). Pearson's correlation coefficients of MitoTracker Orange CMTM Ros and DCF fluorescent labelling colocalization in MII oocytes obtained after IVM culture in presence of VB and vitrified/warmed (panel (c)). Numbers of analyzed oocytes per group are indicated at the bottom of each histogram. (a) One-way ANOVA: NS. (b) One-way ANOVA: a,b: *P* < 0.001. (c) One-way ANOVA: NS.

Concerning mt activity, no significant difference was observed in the comparisons between treated and control groups (Figure 4(a), NS). Intracellular ROS levels increased in the group of oocytes treated with the low VB concentration (1.03 *μ*M) compared with controls (Figure 4(b), *P* < 0.001). In vitrified/warmed oocytes, mt/ROS colocalization, expressed as Pearson's correlation coefficient, was not affected by VB addition (Figure 4(c)).

### 3.10. Low Verbascoside Stability in Oocyte IVM Culture Medium

**Figure 5:**
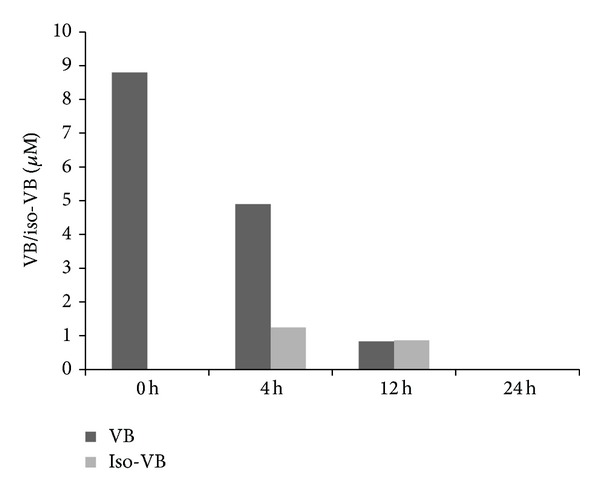
Stability test of VB in oocyte-free IVM medium, incubated at 38.5°C under 5% CO_2_ in air. The figure shows the decrease of VB versus time. isoVB appears after 4 hours of incubation. After 24 hours, both VB and isoVB are not detected.

As shown in Figure 5, after 4 hours of exposure, VB concentration decreased about 55.6% with respect to the initial amount; moreover, the presence of an isomerisation product was recorded. In fact, at this time point, its positional isomer, isoverbascoside (isoVB) of occurred in the proportion of 20% to the total. After 12 h with exposure in oocyte-free medium, further reduction of VB concentration, by 80% with respect to the initial amount, was found. Verbascoside and its positional isomer isoVB, at this time, were present with the same proportions. After 24 hours, no traces of both polyphenols were recovered. No specific degradation products were characterized beyond isoVB.

### 3.11. Rapid Verbascoside Uptake in Prepubertal Lamb OCCs

To evaluate absorptive potential of the OCC, VB accumulation experiments were conducted on two groups of 36 OCCs each, one examined at 30 min and the other examined at 60 min after the start of exposure. Uptake of VB was found to be time dependent and reached the maximum at 30 min after start of exposure. At 100 *μ*M, OCCs accumulated 79.4 nmol of VB/mg of OCCs proteins. It represents approximately 0.2% of absorption of the tested VB. At 60 min, VB accumulated by the OCCs decreased to 19.05 nmol of VB/mg of OCCs proteins.

## 4. Discussion

The highly purified VB from OMWW was previously analyzed for assessing its antioxidant activity and, after brief incubation in HT-29 cell line, it showed antioxidant properties [2]. However, at the typical conditions used in the present study, VB showed an opposite behavior. In fact, this compound had no effect on oocyte nuclear maturation rate. IVM data obtained with media 1 and 2 were kept separated in order to avoid misinterpretation due to the effects of different culture conditions. In both tested media, no significant differences were noticed between VB-treated and control oocytes. Moreover, a reduction of the blastocyst formation rate after exposure to intermediate and high VB doses was found. This result can be considered as a long-term effect of VB or VB derived products on oocyte cytoplasmic competence and consequently on embryo development. These findings lead us to hypothesize that, in our cell system, VB had an oxidatively stressful and toxic effect probably as a consequence of a prolonged exposure time at the tested concentrations or toxicity resulting from oxidative degradation of VB and subsequent generation of H_2_O_2_ as reported for oxidation of phenolic compounds in culture media [60]. This observation is interesting considering that the concentrations used in these studies were reported as being effective in modulating oxidative stress in HT29 cell line after brief (30 min) *in vitro* exposure [2].

In order to investigate the reasons of the observed compromised embryo development, we investigated the bioenergetic potential and oxidative status of oocytes exposed to VB. The supplementation of the IVM medium with VB did not change mt distribution pattern and intracellular ROS localization in prepubertal lamb oocytes. However, in the group of oocytes exposed to the highest VB concentration, it increased mt activity and intracellular ROS levels without affecting mt/ROS colocalization. This finding allowed us to consider that the two fluorescence signals were not overlapping; thus observed ROS increase could be not only due to mt ROS generation, rather it could be also of cytosolic origin, thus indicating a stressed/toxic cytoplasmic condition. In fact, high levels of mt/ROS colocalization were reported as a reliable marker of healthy MII oocytes [40]. In the culture conditions (VB exposure times and tested concentrations) of this study, the ooplasmic bioenergetic/redox status was not improved by VB addition, as confocal analysis stated that VB, at the tested concentrations, failed to behave as an antioxidant compound, a role which was previously demonstrated in other cell line system [2, 3, 6, 7]; rather it demonstrated a prooxidant effect.

To provide further information about the effects of VB on oocyte cytoplasm oxidative status, the activities of two scavenging enzymes, CAT and SOD, in VB exposed oocytes were tested. The assays demonstrated that while SOD activity was not affected, CAT activity significantly increased upon VB addition. This increase could be due to the potential formation of H_2_O_2_ during oxidation of phenolics in cell culture media. Verbascoside stability was observed to be poor in the conditions tested (Figure 5). Oxidative degradation of phenolic compounds has been reported to proceed through auto-oxidative mechanism involving formation of H_2_O_2_ [61, 62]. This increase in peroxides would explain the subsequent increase in CAT activity without an increase in SOD activity. The observed lack of variation of SOD activity may also be related to the fact that, in our experiments, total SOD activity was investigated. It cannot be excluded that the relative ratio between cytosolic and mt SOD could be varied upon VB addition, but further studies are necessary to address this hypothesis. The stimulating effect of VB on CAT activity may also be related to the observed increase in intracellular ROS levels. Moreover, it can be interpreted under the light of current literature. In fact, recent *in vivo* and *in vitro* studies report different effects of VB on the antioxidant enzyme network [6, 63–65]. Other studies show that VB increased the expression of the transcription factor peroxisome proliferator activated receptor *α* (PPAR-*α*) in nuclear extracts of colon cells [4]. Another member of PPARs family, PPAR*γ*, has been shown to activate genes with a peroxisome proliferator response element (PPRE) in their promoter regions [66]. Indeed, the CAT promoter gene is known to contain functional PPAR-*γ* responsive elements, so it is possible that the CAT activity could be regulated by PPAR-*γ* agonists [67, 68]. Based on our results and these observations, it is possible to hypothesize that VB may act by increasing oocyte intracellular ROS levels, both by generating H_2_O_2_ through its own oxidative degradation and by increasing CAT expression levels through multiple mechanisms.

As a further step of the JIVET technology and due to the particular interest of establishing suitable cryopreservation procedures allowing the prepubertal oocyte to be protected against cryodamage, the effects of VB added during IVM on the viability and bioenergetic/oxidative status of MII vitrified/warmed oocytes were evaluated. Exposure to high cryoprotectant concentrations and subphysiological temperature generates in the oocyte a widespread oxidative stress, as reported by Zhao et al. [69]. Moreover, Succu et al. [34] demonstrated that prepubertal oocytes are more susceptible than adult counterparts to cryoinjury during vitrification performing. No effects of VB on cell viability parameters of vitrified/warmed oocytes were found. In order to provide additional information on cytoplasmic condition after vitrification/warming, we investigated the effects of VB on the bioenergy/oxidative status of vitrified/warmed oocytes. Just like in fresh oocytes, VB did not affect mt distribution pattern, ROS localization, and mt activity of vitrified oocytes. However, when used at the lowest tested concentration (1.03 *μ*M) during IVM, it increased ROS levels without affecting mt/ROS colocalization. This finding can be explained considering that, in the case of vitrified oocytes (a more stressed cell system compared to their fresh counterpart), the prooxidant toxic effect of VB could have been strengthened, as supported by the comparison between fresh and vitrified oocytes at 1.03 *μ*M VB (*P* = 0.002).

In the light of obtained results and considering that it is well known that, for every drug, the line between benefit and toxicity is represented by the administered dose, differences in the cellular metabolism, dimension, and number of cells per dish between oocytes and cultured cell lines leading to differences in drug uptake which may result as a beneficial or toxic concentration to the cell were hypothesized. For these reasons, we performed experiments to test (1) VB stability in our culture conditions and (2) its uptake by the OCCs. Determinations of VB stability in an oocyte-free IVM medium at 38.5°C indicated that VB was unstable under our IVM culture conditions, as previously reported [70], also for other polyphenols such as catechin, chlorogenic acid [60, 71–73]. After 4 hours of incubation, an isomerization process occurred, with the appearance of the positional isomer, isoVB. This process is probably related to the medium pH (7.2–7.4) and culture temperature (38.5°C) or to the deacetylation and VB caffeoyl moiety migration under hydrolytic environment of harsh extraction conditions [74]. After 12 hours, the residual percentage of VB and isoVB was 20% and reached zero after 24 hours of incubation. Probably, other products derived from oxidation activity were present but, at this point of the research, no information about these products is available. These data are in agreement with the results obtained from other polyphenols that were also unstable under the incubation conditions used [71]. Moreover, VB uptake experiments demonstrated that the compound was rapidly incorporated into the OCCs in a time-dependent manner with an accumulation efficiency of about 0.2% after 30 min. This observed low level of VB uptake was similar to that reported for HT-29 and Caco-2 human intestinal cells [2, 75] and for other polyphenols such as catechins [76–78] and is consistent with poor *in vivo *VB bioavailability, as reported by Funes et al. [79] following oral administration of lemon verbena extract in rodents and by Cardinali et al., [80] with *ex vivo* experiments. The uptake experiments by OCCs also demonstrated that the amount of VB in our cells was one thousand times higher (in the order of nmol/mg proteins) than that found in the Caco-2 and HT 29 human intestinal cell lines (in the order of pmol/mg proteins) by Cardinali et al. [2, 75]. This result could be consistent with the observed prooxidant activity of VB in the current model. Future experiments should consider lower levels of VB exposure to better emulate intracellular concentrations observed in other cell models and to better understand if lower levels may be able to deliver differential protection against oxidative stress.

Taken together, our data demonstrated that VB, at the conditions used in this study, exerted toxic effects (as it was shown by reduced blastocyst formation) which could be induced by its prooxidant activity. This prooxidant activity could be due to an excessively high tested concentration as observed by uptake data, by prolonged incubation time in culture media (24 hours) used in the present study, in which H_2_O_2_ production is probably induced. Hydrogen peroxide production was already demonstrated in previous studies in which other polyphenols have been used in different cell systems [61, 62, 81]. The H_2_O_2_ production was assessed by Odiatou et al. [81] on the two main olive polyphenols (hydroxytyrosol and oleuropein) incubated in the culture media and was observed as being dependent upon the presence of sodium bicarbonate in the medium. Therefore, polyphenols, normally considered as antioxidant, work as prooxidants, when they are added in the commonly used media, in standard culture conditions at high concentrations, thus producing significant amounts of H_2_O_2_. Even for VB, in the culture medium conditions used in this study, it could be possible to speculate the same behaviour. In addition, the possible presence of H_2_O_2_ in the culture medium could justify the increased oocyte intracellular ROS levels and CAT activity.

## 5. Conclusions

In conclusion, VB, added at *μ*-molar concentrations in a continuative 24 hours IVM exposure protocol, acts as a prooxidant molecule, by impairing oocyte bioenergetic potential and oxidative status and embryo developmental competence of prepubertal lamb oocytes. Applications of this compound in oocyte or embryo culture systems could be developed by evaluating its use at lower concentrations and reduced exposure times.

## Figures and Tables

**Table 1:** Effects of verbascoside on the *in vitro* meiotic maturation of prepubertal lamb oocytes.

Verbascoside concentration (*μ*M)	Number of cultured oocytes	IVM medium type	Nuclear chromatin configuration number (%)			
			GV	MI to TI	MII + PB	Degenerated
0	93	1	9 (9.7)^c^	6 (6.5)	59 (63.4)	19 (20.4)^a^
	111	2	27 (24.3)^d^	17 (15.3)	60 (54.1)	7 (6.3)^b^
	**204**	**(∗)**	**36 (17.6)**	**23 (11.3)**	**119 (58.3)**	**26 (12.7)**

1.03	79	1	11 (13.9)	12 (15.2)	44 (55.7)	12 (15.2)
	118	2	27 (22.9)	21 (17.8)	61 (51.7)	9 (7.6)
	**197**	**(∗)**	**38 (19.3)**	**33 (16.8)**	**105 (53.3)**	**21 (10.7) **

2.06	98	1	12 (12.2)^c^	6 (6.1)^a^	67 (68.4)^c^	13 (13.3)
	116	2	33 (28.4)^d^	21 (18.1)^b^	50 (43.1)^d^	12 (10.3)
	**214**	**(∗)**	**45 (21)**	**27 (12.6)**	**117 (54.7)**	**25 (11.7)**

4.11	98	1	11 (11.2)^c^	13 (13.3)	58 (59.2)	16 (16.3)
	137	2	36 (26.3)^d^	23 (16.8)	63 (46)	15 (10.9)
	**235**	**(∗)**	**47 (20)**	**36 (15.3)**	**121 (51.5)**	**31 (13.2)**

GV: germinal vesicle, M: metaphase, T: telophase, and PB: polar body. *Pooled data of oocytes cultured in medium 1 + medium 2. Chi-square test and multifactor ANOVA test: within each medium type and pooled data, VB-treated versus controls: NS; between media, within each VB concentration: ^a,b^ *P* < 0.05; ^c,d^ *P* < 0.001.

**Table 2:** Effects of verbascoside on cleavage and blastocyst developmental rates of prepubertal lamb oocytes after IVF and IVP.

Verbascoside concentration (*μ*M)	Number of MII oocytes	Number of cleaved embryos (%)		Number of blastocysts (%)	
		24 h after-IVF	30 h after-IVF	At day 7	At day 8
0	200	76 (38)	150 (75)	28 (18.7)^a^	36 (24)^a^
1.03	240	103 (42.9)	189 (78.7)	38 (20.1)	59 (31.2)
2.06	220	77 (35)	156 (70.9)	18 (11.5)	18 (11.5)^b^
4.11	240	93 (38.7)	185 (77.1)	11 (5.9)^b^	22 (11.9)^b^

Chi-square test: ^a,b^ *P* < 0.05.

**Table 3:** Effects of verbascoside on the mitochondrial distribution pattern of *in vitro* matured prepubertal lamb oocytes.

Verbascoside concentration (*μ*M)	Number of MII oocytes	Mitochondrial distribution number (%)					
		Homogeneous	Heterogeneous				Abnormal
			Total (1 + 2 + 3)	Perinuclear/ pericortical (1)	Peri-nuclear (2)	Peri-cortical (3)	
0	30	16 (53.3)	14 (46.7)	0 (0)	2 (6.7)	12 (40)	0 (0)
1.03	29	15 (51.7)	12 (41.4)	1 (3.4)	2 (6.9)	9 (31)	2 (6.9)
2.06	25	16 (64)	9 (36.0)	1 (4)	2 (8)	6 (24)	0 (0)
4.11	29	13 (44.8)	16 (55.2)	0 (0)	2 (6.9)	14 (48.3)	0 (0)

Chi-square test: NS.

**Table 4:** Postvitrification/warming viability of prepubertal lamb oocytes matured *in vitro* in presence of verbascoside.

Verbascoside concentration (*μ*M)	Number of MII oocytes vitrified/warmed	Viability number (%)
0	55	28 (50.9)
1.03	41	28 (68.3)
2.06	55	37 (67.3)
4.11	58	29 (50.0)

Chi-square test: NS.

**Table 5:** Post vitrification/warming mitochondrial distribution pattern of prepubertal lamb MII oocytes obtained after IVM in presence of verbascoside.

Verbascoside concentration	Number of MII oocytes	Mitochondrial distribution pattern number (%)					
		Homogeneous	Heterogeneous				Abnormal
			Total (1 + 2 + 3)	Peri-nuclear/peri-cortical (1)	Peri-nuclear (2)	Peri-cortical (3)	
0 (control)	15	8 (53.3)	7 (46.7)	0 (0)	2 (13.3)	5 (33.3)	0 (0)
1.03	18	11 (61.1)	6 (33.3)	2 (11.1)	1 (5.6)	3 (16.7)	1 (5.6)
2.06	19	14 (73.7)	5 (26.3)	0 (0)	0 (0)	5 (26.3)	0 (0)
4.11	14	10 (71.4)	4 (28.6)	0 (0)	2 (14.3)	2 (14.3)	0 (0)

Chi-square test: NS.
